# Combination HIV Prevention Strategies Among Montreal Gay, Bisexual, and Other Men Who Have Sex with Men in the PrEP Era: A Latent Class Analysis

**DOI:** 10.1007/s10461-020-02965-4

**Published:** 2020-07-09

**Authors:** Carla M. Doyle, Mathieu Maheu-Giroux, Gilles Lambert, Sharmistha Mishra, Herak Apelian, Marc Messier-Peet, Joanne Otis, Daniel Grace, Trevor A. Hart, David M. Moore, Nathan J. Lachowsky, Joseph Cox, the Engage Study Team

**Affiliations:** 1 Department of Epidemiology, Biostatistics, and Occupational Health, McGill University, Montreal, QC Canada; 2 Direction Régionale de Santé Publique de Montréal, Montreal, QC Canada; 3 Department of Medicine, St. Michael’s Hospital, University of Toronto, Toronto, ON Canada; 4 Institute of Medical Sciences, University of Toronto, Toronto, ON Canada; 5 Institute of Health Policy Management and Evaluation, Dalla Lana School of Public Health, University of Toronto, Toronto, ON Canada; 6 Département de Sexologie, Université du Québec à Montréal, Montreal, QC Canada; 7 Dalla Lana School of Public Health, University of Toronto, Toronto, ON Canada; 8 Department of Psychology, Ryerson University, Toronto, ON Canada; 9 Division of Social & Behavioural Sciences, Dalla Lana School of Public Health, University of Toronto, Toronto, ON Canada; 10 BC Centre for Excellence in HIV/AIDS, Vancouver, BC Canada; 11 Faculty of Medicine, University of British Columbia, Vancouver, BC Canada; 12 School of Public Health and Social Policy, University of Victoria, Victoria, BC Canada; 13 Clinical Outcomes Research and Evaluation, Research Institute - McGill University Health Centre, Montreal, QC Canada

**Keywords:** Combination HIV prevention, HIV prevention strategies, Men who have sex with men, Pre-exposure prophylaxis, Latent class analysis

## Abstract

Pre-exposure prophylaxis (PrEP) became publicly available in Quebec for gay, bisexual and other men who have sex with men (GBM) in 2013. We used baseline data from Engage, a cohort of GBM recruited by respondent-driven sampling, to examine patterns of combination HIV prevention use among Montreal GBM since PrEP became available. Latent class analysis, stratified by HIV status, was used to categorize GBM by self-reported use of biomedical and behavioural prevention strategies. Correlates of resulting classes were identified using multinomial logistic regression. Among HIV-negative/unknown GBM (n = 968), we identified four classes: *low use of prevention* (32%), *condoms* (40%), *seroadaptive behaviour* (21%), and *biomedical* (including PrEP; 7%). Those using prevention (*condoms*, *seroadaptive behaviour*, and *biomedical*) had a higher number of anal sex partners and were more likely to report a recent sexually transmitted infection diagnosis*.* GBM using biomedical prevention also had a higher level of formal education. Among GBM living with HIV (n = 200), we identified three classes: *mainly antiretroviral treatment (ART) with viral suppression* (53%), *ART with viral suppression and condoms* (19%), and *ART with viral suppression and seroadaptive behaviour* (18%). Again, the number of anal sex partners was higher among those using condoms and seroadaptive behaviours. Our findings show antiretroviral-based prevention, either alone or in combination with other strategies, is clearly a component of the HIV prevention landscape for GBM in Montreal. Nevertheless, PrEP uptake remains low, and there is a need to promote its availability more widely.

**Electronic supplementary material:**

The online version of this article (10.1007/s10461-020-02965-4) contains supplementary material, which is available to authorized users.

## Introduction

Gay, bisexual, queer, and other men who have sex with men (GBM), including transmen, bear a disproportionate burden of HIV in Canada [1]. The most recent national estimate suggests GBM accounted for 41% of diagnosed HIV infections in 2018 [1]. In the province of Quebec, which had the second-largest proportion of HIV diagnoses in the country [1], GBM comprised 58% of those occurring in males, and the majority were in the Montreal metropolitan area [2]. In 2017, Montreal announced it would become the first UNAIDS Fast-Track city in Canada [3] and committed to ending the HIV/AIDS epidemic by 2030 [4]. With this renewed drive towards HIV elimination, it is necessary to assess current strategies used for HIV prevention by GBM in Montreal and to understand the factors associated with prevention use to devise appropriate prevention policy and programming.

Combination HIV prevention hinges upon the concurrent use of behavioural, biomedical, and structural prevention strategies to reduce HIV transmission [5]. By promoting a targeted set of prevention strategies working synergistically on multiple levels (e.g. individual, partnership, and population), combination prevention programs can have an improved impact on transmission [6, 7]. At the individual level, combination prevention can involve simultaneously practicing more than one strategy, or adopting one during a specific time or context, while adopting alternative strategies in others. Early in the epidemic, the conventional biomedical HIV prevention strategy promoted for GBM was condom use during anal sex [8–10]. However, GBM have come to use and combine additional strategies over time to prevent the acquisition and transmission of HIV. These include behavioural seroadaptive strategies, such as serosorting and strategic positioning [11–17], and other biomedical prevention strategies such as HIV testing, antiretroviral treatment (ART) [18–21], post-exposure prophylaxis (PEP) [22, 23], and pre-exposure prophylaxis (PrEP) [24, 25].

The greatest potential for HIV elimination [26] lies in the use of antiretroviral medications for prevention through ART (a combination of antiretrovirals for treatment among people living with HIV) and PrEP (antiretrovirals for people uninfected with HIV). ART decreases HIV viral load to undetectable levels. Studies have shown that those on ART with an undetectable viral load do not transmit the virus to their sexual partners [18–21], giving rise to the notion of Undetectable = Untransmissible (U = U) [27] and ART as prevention. PrEP is also highly effective in preventing HIV acquisition [24, 25]. The evolution of these two strategies has led to the HIV “status-neutral” approach to prevention programming, which emphasizes the engagement of individuals into clinical HIV care, regardless of HIV status [28]. In this way, both people living with HIV and HIV-negative individuals similarly enter a cascade of care, be-it for treatment or prevention. Such an approach is now fundamental to combination HIV prevention, with U = U and PrEP especially being prioritized by elimination efforts [29–31].

Previous Canadian studies have examined the use of prevention strategies among GBM [32, 33], but did not always include GBM living with HIV [32]. Further, these were conducted before PrEP became formally recommended. In 2013, Quebec became the first Canadian province to publicly reimburse antiretroviral medication (Truvada) for PrEP for at-risk GBM [34]; it remained the only one to do so until 2017 when other provinces followed. Consequently, combination HIV prevention including the use of PrEP-related strategies has yet to be understood in Quebec and elsewhere in Canada.

Latent class analysis (LCA) is a statistical method that identifies underlying patterns in data to uncover groupings (latent classes) of individuals that are similar according to particular characteristics [35, 36]. Many studies have used this method to understand various characteristics and behaviours related to HIV, including sociodemographic and sexual risk practices [37], substance use [38–40] and other syndemic factors [41], as well as prevention use among GBM in Canada [32, 33] and elsewhere [42, 43]. Given the number of existing strategies, LCA can be useful for discerning relevant patterns in prevention use. Rather than using pre-determined categories that may not meaningfully describe the reality, LCA considers all potential combinations of strategies and simplifies this complexity by identifying the most frequently occurring response patterns in the data [35, 36]. For a full picture, we can further determine the attributes of individuals within each class to explain who utilizes particular types of prevention. Factors previously found to be associated with prevention use range across many dimensions, such as sociodemographic (including social support [44, 45]), sexual health (including sexual behaviours, relationships, attitudes, and sexually transmitted or blood-borne infections [STBBIs] [32, 46]), substance use, and other health-related factors (including mental health [44, 47, 48]).

We aimed to describe the prevention strategies currently practiced by GBM in Montreal, the second-largest Canadian city and epicentre of the HIV epidemic in Quebec. Our objectives were to (1) examine patterns in the use of prevention strategies among HIV-negative GBM and GBM living with HIV in Montreal distinctively and (2) describe the potentially important sociodemographic, behavioural and health-related factors associated with observed patterns. This assessment could aid policymakers in identifying prevention gaps and inform future responses to ensure prevention uptake is in-line with elimination needs.

## Methods

### Study Population

*Engage* is a prospective cohort study of GBM in Montreal, Toronto, and Vancouver. We included the baseline data of participants from Montreal [46], where recruitment occurred between February 2017 and June 2018. Cisgender and transgender men aged ≥ 16 years were eligible to participate if they had sex with another man in the past 6 months (P6M), resided in the greater Montreal area, could read in French or English and consented to participate. All participants completed a computer-assisted questionnaire in French or English and underwent HIV and other STBBI testing with a study nurse.

Participants were recruited using respondent-driven sampling (RDS) [49]. Initial participants were purposively selected to initiate recruitment chains, with successive participants distributing up to six coupons to recruit peers. Participants were compensated $50 for their initial enrollment and $15 for each successful recruit. RDS results are presented following STROBE-RDS guidelines [50]; see Online Appendix I.

Ethics approval for the Montreal Engage site was obtained from the Research Institute of the McGill University Health Centre.

### Use of HIV Prevention Strategies by HIV Status

Self-reported HIV status, defined by the self-reported result (in the questionnaire and to the nurse) of their last HIV test, was used as this captured each participant’s awareness of their serostatus at enrollment, and this would have influenced prior sexual behaviours and prevention use. Those who reported never tested, unsure if ever tested, or never receiving their last result were considered not to know their HIV status and were assumed to have similar sexual behaviours to HIV-negative men. Thus, we dichotomized HIV status to HIV-negative/unknown and HIV-positive [33, 51, 52]. Those that self-reported as HIV-negative/unknown in the questionnaire but as living with HIV to the study nurse were considered HIV-positive (n = 11) and excluded from these analyses, as the questionnaire’s skip pattern resulted in missing information on the prevention strategies for people living with HIV. As the use of HIV prevention differs according to HIV serostatus, we considered two sets of strategies: (1) those among HIV-negative/unknown and (2) those among individuals living with HIV. Within each, we included biomedical (testing, condom and antiretroviral-based) and behavioural (seroadaptive) prevention strategies, as these are most proximal to HIV acquisition and transmission [53]. All measures of prevention were self-reported (see Online Appendix II for survey questions).

#### HIV-Negative/Unknown Individuals

Measures concerning prevention of HIV acquisition among HIV-negative/unknown GBM included: recent HIV testing (P6M); consistent condom use (*always used condoms for anal sex*; P6M); any PEP use (ever); any PrEP use (P6M); any strategic positioning (*positioned as the top [insertive partner] for anal sex to prevent acquiring HIV*; P6M); any serosorting (*condomless sex with known HIV-negative men to prevent acquiring HIV*; P6M); and any viral load sorting (*condomless sex with HIV-positive men who have an undetectable viral load*; P6M).

#### Individuals Living with HIV

Measures concerning prevention of HIV transmission by GBM living with HIV included: consistent condom use (as above; P6M); ART with viral suppression (self-reported undetectable viral load [< 50 copies/mL]; current); any strategic positioning *(positioned as the bottom [receptive partner] for anal sex to prevent transmitting HIV*; P6M); any serosorting (*condomless sex with known HIV-positive men to prevent transmitting HIV*; P6M); any PrEP-use sorting (*condomless sex with HIV-negative men using PrEP*; P6M).

### Statistical Analyses

We described sample characteristics with and without RDS-adjustment.

#### Latent Class Analyses

We used LCA to empirically categorize participants into classes based on their use of prevention strategies. All LCA models were stratified by self-reported HIV status and included corresponding indicators for the use of prevention strategies (defined above).

An assumption of LCA is conditional independence [35, 54]. Among the HIV-negative/unknown prevention strategies, by definition, separate indicators for serosorting and viral load sorting would likely be conditionally dependent, as would separate indicators for serosorting and PrEP-use sorting among the HIV-positive prevention strategies. To relax the assumption of conditional independence, a single item for serosorting or viral load sorting (yes to either vs. no to both) was used in the HIV-negative/unknown LCA models, resulting in a measure of having any condomless sex with GBM that could reduce the risk of HIV acquisition as a seroadaptive strategy. In the HIV-positive models, a four-level joint item indicator [55] for serosorting and/or PrEP-use sorting was used (using both, PrEP-use sorting and not serosorting, serosorting and not PrEP-use sorting, and neither). Conditional dependence might also arise if participants responded consistently to the survey items for condom use, serosorting, viral load sorting, and PrEP-use sorting (Online Appendix II). To assess the validity of the conditional independence assumption, we examined the bivariate residuals between pairs of strategies [56]. Aside from the abovementioned exception of serosorting and/or PrEP-use sorting, binary indicators were used in the LCA models.

The optimal number of latent classes was based on the interpretability of those classes and model fit criterion; mainly Bayes Information Criterion (BIC), Akaike Information Criterion (AIC), and entropy [57]. Specifically, models with 1–5 classes and 1–3 classes were investigated for the HIV-negative/unknown and the HIV-positive models, respectively. Class profiles were assessed based on the resulting conditional probabilities and labels were assigned qualitatively according to the main and defining strategies used. Class sizes were adjusted using the RDS-II-estimator [58], which applies inverse probability of sampling weights proportional to participant network size.

#### Correlates of Class Membership

Factors known [32, 33, 44, 45, 59–61] or hypothesized to be associated with use of prevention strategies that were well-measured, had few missing data (< 5%), and had sufficient cell counts (≥ 5) when stratified by class (HIV-positive models; see Table 5), were selected a priori and included as covariates in multivariable regression models. Sociodemographic factors were: age, sexual orientation, ethnicity, education and social time spent with gay/bisexual guys. Sexual health and related factors included: unknown HIV status (HIV-negative models), STBBI diagnosis in the past 12 months (P12M), number of anal sex partners (P6M), HIV status of main partner (HIV-negative models; we used having a main partner in HIV-positive models due to small cell counts), perceived risk of acquiring/transmitting HIV, and HIV treatment optimism (measured by the HIV Treatment Optimism-Skepticism Scale [62], which ranges from 0 to 36, with higher scores indicating higher optimism in ART). Other health-related factors included: having a regular health care provider and perceived mental health (P6M). Finally, substance use factors included: use of crack or cocaine (P6M), use of other drugs, and alcohol misuse (measured by the Alcohol Use Disorders Identification Test, consumption questions [AUDIT-C] [63], which ranges from 0 to 12, where higher scores indicate higher risk of alcohol affecting one’s health and safety; scores were dichotomized at 4, the optimal cut point for identifying alcohol dependence in men [64]).

#### Multinomial Logistic Regression Modelling

Univariable and multivariable multinomial logistic regression models stratified by self-reported HIV status assessed the factors associated with each class. Class membership was assigned by modal assignment, according to the class each individual had the highest probability of belonging to. The referent class was chosen based on size and consistency between the HIV-negative/unknown and HIV-positive models. As there was very little missing data amongst the factors modelled (≤ 3.1%), complete case analyses were performed. RDS weights were not included, as these may be unwarranted in regression modelling [65]. Robust standard errors were used to account for clustering by each recruiter within the referral chain.

All analyses were conducted in the R statistical software using the *poLCA*, *mlogit*, and *sandwich* packages [66–68].

## Results

### Study Population

Engage enrolled 1168 participants in Montreal: 200 (17%) self-reported as living with HIV and 863 (74%) as HIV-negative; the remaining 105 (9%) did not know their HIV status (total of 968 HIV-negative/unknown participants). Table 1 summarizes the participant sociodemographic characteristics. The mean age of participants was 38 years [standard deviation (SD) 14]. The majority identified as a man (94%) and as gay (86%). Approximately half of the sample identified as French Canadian, 65% had a post-high school diploma or higher, and 43% had an annual income of CAD 30,000 or more. Over half (57%) did not have a main partner at the time of participation. The mean number of anal sex partners (P6M) was 7 (SD 15), and 31% of participants reported an STBBI diagnosis (P12M).

**Table 1:** Unadjusted and RDS-II adjusted estimates of socio-demographic characteristics of the Engage-Montreal study participants, 2017–2018 (n = 1168)^a^

Characteristic	Overall (n = 1168)		HIV-negative/unknown (n = 968)		HIV-positive (n = 200)	
	n (%)	RDS-II weighted % (95% CI)	n (%)	RDS-II weighted % (95% CI)	n (%)	RDS-II weighted % (95% CI)
Age (mean)	38 (SD 14)	38 (36–39)	36 (SD 13)	36 (35–37)	49 (SD 11)	50 (48–52)
Gender: man^b^	1101 (94%)	92% (89–95%)	912 (94.2%)	92% (88–95%)	189 (95%)	93% (89–98%)
Sexual orientation: gay^c^	1009 (86%)	81% (77–85%)	827 (85%)	80% (75–84%)	182 (91%)	90% (81–98%)
Ethnicity						
French Canadian	605 (52%)	44% (39–49%)	477 (50%)	43% (37–48%)	128 (66%)	57% (45–69%)
English Canadian	111 (10%)	10% (7–13%)	89 (9%)	9% (6–12%)	22 (11%)	17% (7–26%)
European	156 (14%)	15% (11–18%)	142 (15%)	16% (12–19%)	14 (7%)	8% (1–14%)
Other	283 (25%)	31% (26–36%)	253 (26%)	33% (27–38%)	30 (16%)	18% (10–27%)
Education: post-high school diploma or higher	757 (65%)	58% (53–63%)	663 (69%)	60% (55–65%)	94 (47%)	42% (30–53%)
Annual income: $30,000 CAD or more	500 (43%)	33% (29–38%)	435 (45%)	34% (29–38%)	65 (33%)	32% (21–43%)
Social time spent with gay/bi guys: 50% or more	521 (46%)	33% (28–38%)	432 (46%)	32% (27–37%)	89 (46%)	38% (27–48%)
HIV status: unknown	105 (9%)	13% (10–16%)	105 (11%)	15% (11–18%)	–	–
STBBI diagnosis in the past 12 months	359 (31%)	26% (21–31%)	255 (26%)	23% (18–28%)	104 (52%)	44% (32–56%)
Mean number of anal sex partners in the past 6 months	7 (SD 15)	5 (3–7)	7 (SD 15)	5 (3–7)	10 (SD 16)	6 (4–8)
HIV status of main partner						
No main partner	662 (57%)	56% (51–61%)	538 (56%)	56% (50–61%)	124 (62%)	56% (44–68%)
Unknown/uncertain	121 (10%)	12% (8–15%)	113 (12%)	12% (9–16%)	8 (4%)	6% (0–15%)
HIV-negative	328 (28%)	28% (23–32%)	287 (30%)	29% (24–34%)	41 (21%)	22% (12–32%)
HIV-positive	57 (5%)	5% (3–7%)	30 (3%)	3% (1–5%)	27 (14%)	16% (9–24%)
Perceived risk of acquiring/transmitting HIV	187 (17%)	19% (15–23%)	171 (18%)	20% (16–25%)	16 (9%)	8% (2–15%)
HIV Optimism-Skepticism Scale^d^ (mean)	17 (SD 6)	16 (16–17)	16 (SD 5)	16 (15–17)	20 (SD 6)	20 (18–22)
Currently have a health care provider	786 (67%)	60% (55–65%)	599 (62%)	54% (49–60%)	187 (94%)	95% (90–100%)
Perceived mental health in the past 6 months						
Excellent/very good	552 (49%)	47% (42–53%)	459 (48%)	46% (40–52%)	93 (48%)	56% (44–68%)
Good	332 (29%)	28% (24–33%)	278 (29%)	30% (24–35%)	54 (28%)	19% (10–28%)
Fair/poor	258 (23%)	24% (20–29%)	211 (22%)	24% (19–29%)	47 (24%)	25% (14–37%)
Drug use: crack or cocaine in the past 6 months	318 (27%)	24% (20–29%)	269 (28%)	24% (20–28%)	49 (25%)	28% (18–39%)
Drug use: other drugs in the past 6 months^e^	538 (46%)	36% (32–41%)	429 (44%)	35% (30–40%)	109 (56%)	43% (31–54%)
Alcohol misuse: AUDIT-C ≥ 4^f^	685 (60%)	55% (50–60%)	605 (63%)	57% (51–62%)	90 (46%)	42% (30–53%)

*RDS* respondent driven sampling, *CI* confidence interval, *SD* standard deviation, *STBBI* sexually transmitted or blood borne infection, *AUDIT-C* alcohol use disorders identification test, consumption questions

^a^RDS-II weights are inverse probability of sampling weights that are proportional to participant network size

^b^Gender was defined as man versus other. The other terms used to describe one’s gender included: transman, gender queer/gender non-conforming, and two-spirit

^c^Sexual orientation was defined as gay versus other. The other terms used to describe one’s sexual orientation included: bisexual, straight, queer, questioning, asexual, pansexual and two-spirit

^d^The HIV Treatment Optimism-Skepticism Scale [62] includes items related to the efficacy of antiretrovirals for both HIV treatment and reduced infectiousness. The scale ranges from 0 to 36, where higher scores indicate higher optimism in antiretroviral treatment. Scores were dichotomized at the optimal cut point for identifying alcohol dependence in men [64]: ≥ 4 vs. lower

^e^Other drugs included any of ecstasy, crystal methamphetamine, mephedrone, speed, poppers, gamma hydroxybutyrate (GHB), lysergic acid diethylamide (LSD), and ketamine

^f^Alcohol misuse was measured by the Alcohol Use Disorders Identification Test, consumption questions (AUDIT-C), a screening tool for alcohol abuse, dependence, or heavy drinking [63]. The AUDIT-C Scale ranges from 0 to 12, where higher scores indicate higher risk of alcohol affecting one’s health and safety

### Latent Class Analysis: HIV-Negative/Unknown

**Fig. 1:**
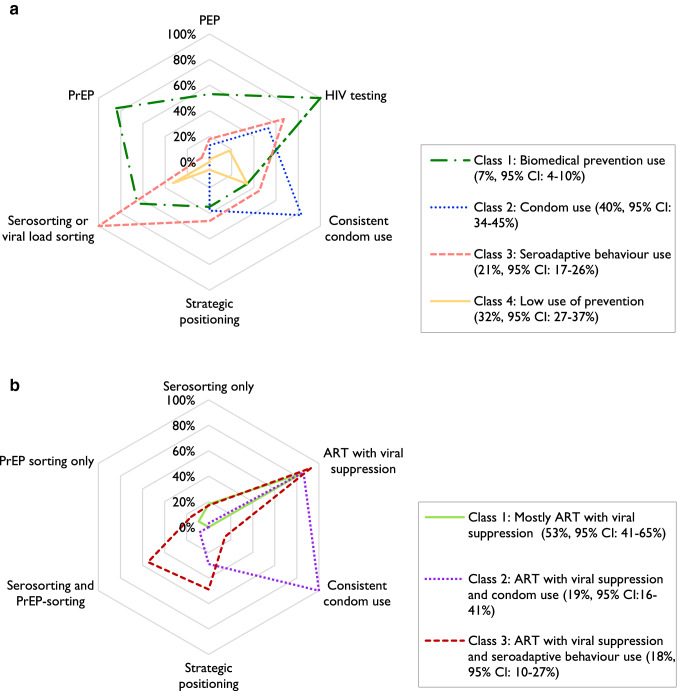
**a** Spider plot of estimated item response probabilities of self-reported use of HIV prevention strategies among the HIV-negative/unknown participants of the Engage-Montreal study, 2017–2018 (n = 968): four class model. **b** Spider plot of estimated item response probabilities of self-reported use of HIV prevention strategies among the HIV-positive participants of the Engage-Montreal study, 2017–2018 (n = 200): three class model

Among the HIV-negative/unknown participants, a four-class model was selected based on fit statistics (Table S2) and model interpretability. None of the bivariate residuals (Table S3) violated the conditional independence assumption. The results are displayed in Fig. 1; Table 2 contains the exact item response probabilities (RDS-weighted class sizes are presented in both; Table S4 provides unweighted estimates).

**Table 2:** Estimated item response probabilities of self-reported use of HIV prevention strategies among the HIV-negative/unknown participants of the Engage-Montreal study, 2017–2018 (n = 968): 4 class model^a^

Variable	Class 1: Biomedical prevention use n = 113 (7%, 95% CI 4–10%)	Class 2: Condom use n = 341 (40%, 95% CI 34–45%)	Class 3: Seroadaptive behaviour use n = 241 (21%, 95% CI 17–26%)	Class 4: Low use of prevention n = 273 (32%, 95% CI 27–37%)
HIV testing	100%	53%	67%	18%
PrEP	84%	0%	7%	0%
PEP	53%	13%	18%	2%
Consistent condom use	34%	83%	45%	34%
Strategic positioning	35%	38%	46%	6%
Serosorting or viral load sorting	65%	0%	100%	33%

*CI* confidence interval, *PrEP* pre-exposure prophylaxis, *PEP* post-exposure prophylaxis

^a^RDS-II adjusted class sizes (%) and 95% confidence intervals are presented. RDS-II weights are inverse probability of sampling weights that are proportional to participant network size

Class 1 was labelled as *biomedical prevention use* (n = 113), Class 2 as *condom use* (n = 341), Class 3 as *seroadaptive behaviour use* (n = 241), and Class 4 as *low use of prevention* (n = 273). Those in *biomedical prevention use*, the smallest class, had the highest probability of recent PrEP use (84% in P6M), ever using PEP (53%), and recent HIV testing (100% in P6M); these participants also engaged in serosorting or viral load sorting (65%). *Condom use,* the largest class, consisted of participants with high levels of consistent condom use (83%) and recent HIV testing (53% in P6M). Those in the *seroadaptive behaviour use* class engaged in serosorting or viral load sorting (100%), had a recent HIV test (67% in P6M), reported consistent condom use (45%), and strategic positioning (46%). Lastly, among the *low use of prevention* class, the highest probability of using any one method corresponded to condom use (34%), followed by having performed serosorting or viral load sorting (33%); the remaining prevention strategies assessed had low probabilities.

### Latent Class Analysis: HIV-Positive

Among the participants living with HIV, a three-class model was selected (Table S5). None of the bivariate residuals violated the conditional independence assumption (Table S6). The results are displayed in Fig. 1; Table 3 contains the exact item response probabilities (RDS-weighted class sizes are presented in both; Table S7 provides unweighted estimates).

**Table 3:** Estimated item response probabilities of self-reported use of HIV prevention methods among the HIV-positive participants of the Engage-Montreal study, 2017–2018 (n = 200): 3 class model

Variable	Class 1: Mostly ART with viral suppression n = 87 (53%, 95% CI 41–65%)	Class 2: ART with viral suppression and condom use n = 46 (19%, 95% CI 16–41%)	Class 3: ART with viral suppression and seroadaptive behaviour use n = 67 (18%, 95% CI 10–27%)
ART with viral suppression	84%	86%	93%
Consistent condom use	0%	100%	15%
Strategic positioning	0%	29%	49%
Serosorting and/or PrEP sorting:			
Both	0%	8%	55%
PrEP sorting only	9%	0%	16%
Serosorting only	18%	3%	17%

*ART* antiretroviral treatment, *CI* confidence interval, *PrEP* pre-exposure prophylaxis

RDS-II adjusted class sizes (%) and 95% confidence intervals are presented. RDS-II weights are inverse probability of sampling weights that are proportional to participant network size

We labelled Class 1 as *mostly ART with viral suppression* (n = 87), Class 2 as *ART with viral suppression and condom use* (n = 46), and Class 3 as *ART with viral suppression and seroadaptive behaviour use* (n = 67). Among all classes, the proportion reporting a suppressed viral load was very high (84–93%). *Mostly ART with viral suppression,* the largest class, consisted of virally suppressed participants (84% probability) with a low or null probability of using any other prevention strategy of interest. Those in the *ART with viral suppression and condom use* class consistently used condoms (100% probability). Lastly, in the *ART with viral suppression and seroadaptive behaviour use* class, participants performed serosorting and/or PrEP sorting, with a 55% probability of performing both, 16% probability of PrEP-sorting only, and 17% probability of serosorting only. This class also had a high probability (49%) of using strategic positioning.

### Multinomial Logistic Regression Models

#### HIV-Negative/Unknown Individuals

The referent class used was *low use of prevention*. The multivariable model indicates those in the *biomedical prevention use, condom use, and seroadaptive behaviour use* classes were more likely to report an increased number of anal sex partners (P6M) across all categories and having had an STBBI diagnosis (P12M) (Table 4; qualitative overview in Table S8). The *seroadaptive behaviour use* class members were further distinguished by being less likely to have an unknown HIV status (aOR 0.5, 95% CI 0.2–1.0). Those in the *biomedical prevention use* class were more likely to have obtained a post-high school diploma or higher (aOR 2.8, 95% CI 1.5–5.3) and were also likely to have a main partner whose HIV-status is positive (aOR 3.4, 95% CI 1.0–11.4), perceived themselves less at risk of HIV (aOR 0.5, 95% CI 0.2–1.0), and had a higher HIV Optimism-Skepticism score (aOR 1.1, 95% CI 1.0–1.2).

**Table 4:** Univariable and multivariable multinomial logistic regression model results assessing factors associated with latent class membership among the HIV-negative/unknown participants of the Engage-Montreal study, 2017–2018 (n = 968)^a^

Variable	Class 1: Biomedical prevention use		Class 2: Condom use		Class 3: Seroadaptive behaviour use	
	OR (95% CI)	aOR (95% CI)	OR (95% CI)	aOR (95% CI)	OR (95% CI)	aOR (95% CI)
Age ≤ 30	0.8 (0.5, 1.3)	0.7 (0.4, 1.3)	0.9 (0.7, 1.3)	0.8 (0.6, 1.2)	1.2 (0.8, 1.7)	0.9 (0.6, 1.4)
Sexual Orientation: gay^b^	3.6 (1.7, 7.9)	0.8 (0.3, 2.0)	1.6 (1.1, 2.4)	1.3 (0.8, 2.2)	2.5 (1.5, 4.0)	1.3 (0.7, 2.4)
Ethnicity						
French Canadian	1.00 (referent)	1.00 (referent)	1.00 (referent)	1.00 (referent)	1.00 (referent)	1.00 (referent)
English Canadian	0.6 (0.3, 1.4)	0.6 (0.2, 1.7)	0.9 (0.5, 1.6)	1.2 (0.7, 2.0)	1.1 (0.6, 2.0)	0.9 (0.5, 1.9)
European	1.9 (0.9, 4.0)	1.1 (0.4, 3.0)	2.5 (1.5, 4.1)	2.5 (1.4, 4.5)	2.9 (1.7, 5.1)	1.9 (1.1, 3.5)
Other	1.6 (0.9, 2.7)	1.1 (0.6, 2.2)	2.3 (1.5, 3.4)	2.4 (1.5, 3.8)	1.7 (1.1, 2.7)	1.4 (0.8, 2.4)
Education: post-high school diploma or higher	3.0 (1.8, 5.3)	2.8 (1.5, 5.3)	1.4 (1.0, 2.0)	1.1 (0.8, 1.6)	2.1 (1.4, 2.9)	1.4 (0.9, 2.1)
Social time spent with gay/bi guys: 50% or more	1.8 (1.2, 2.7)	0.8 (0.5, 1.4)	0.8 (0.6, 1.1)	0.8 (0.6, 1.1)	1.7 (1.2, 2.4)	1.5 (1.0, 2.2)
HIV status: unknown	0.3 (0.1, 0.7)	0.6 (0.2, 1.9)	0.7 (0.4, 1.0)	0.8 (0.5, 1.5)	0.3 (0.1, 0.7)	0.5 (0.2, 1.0)
STBBI diagnosis in the past 12 months	11.0 (6.7, 18.1)	4.9 (2.6, 9.2)	1.7 (1.1, 2.6)	1.6 (1.0, 2.7)	4.3 (2.8, 6.8)	3.2 (1.9, 5.4)
Number of anal sex partners in the past 6 months						
0–1	1.00 (referent)	1.00 (referent)	1.00 (referent)	1.00 (referent)	1.00 (referent)	1.00 (referent)
2–3	4.5 (1.5, 13.3)	6.5 (2.1, 20.2)	2.7 (1.8, 4.1)	2.6 (1.7, 4.1)	4.6 (2.8, 7.5)	4.2 (2.4, 7.1)
4–5	12.3 (3.9, 38.2)	13.1 (3.9, 43.6)	2.9 (1.6, 5.1)	2.5 (1.4, 4.7)	7.9 (4.3, 14.6)	6.1 (3.2, 11.7)
6+	67.9 (26.6, 173.4)	63.5 (23.1, 174.7)	3.2 (2.1, 5.0)	2.7 (1.6, 4.6)	10.5 (6.2, 17.7)	6.4 (3.6, 11.4)
HIV status of main partner						
No main partner	1.00 (referent)	1.00 (referent)	1.00 (referent)	1.00 (referent)	1.00 (referent)	1.00 (referent)
Unknown/uncertain	0.2 (0.1, 0.6)	0.3 (0.1, 1.0)	0.7 (0.4, 1.1)	0.6 (0.4, 1.1)	0.7 (0.4, 1.3)	0.9 (0.5, 1.6)
HIV-negative	0.6 (0.4, 1.1)	1.1 (0.6, 2.0)	0.6 (0.4, 0.8)	0.5 (0.4, 0.8)	1.0 (0.7, 1.5)	1.3 (0.8, 2.0)
HIV-positive	2.2 (0.8, 5.6)	3.4 (1.0, 11.4)	0.4 (0.1, 1.1)	0.5 (0.1, 1.5)	0.7 (0.3, 2.1)	0.8 (0.3, 2.4)
Perceived risk of acquiring HIV	1.0 (0.6, 1.8)	0.5 (0.2, 1.0)	0.9 (0.6, 1.4)	0.7 (0.4, 1.2)	1.8 (1.2, 2.7)	1.3 (0.8, 2.2)
HIV Optimism-Skepticism Scale^c^	1.1 (1.1, 1.2)	1.1 (1.0, 1.2)	1.0 (0.9, 1.0)	1.0 (0.9, 1.0)	1.0 (1.0, 1.1)	1.0 (1.0, 1.0)
Currently have a health care provider	2.1 (1.3, 3.4)	2.1 (1.1, 3.9)	0.9 (0.6, 1.2)	1.1 (0.7, 1.5)	0.9 (0.6, 1.2)	1.0 (0.6, 1.4)
Perceived mental health in the past 6 months						
Excellent/very good	1.00 (referent)	1.00 (referent)	1.00 (referent)	1.00 (referent)	1.00 (referent)	1.00 (referent)
Good	1.1 (0.7, 1.8)	1.6 (0.8, 3.0)	0.8 (0.5, 1.1)	0.8 (0.5, 1.2)	1.2 (0.8, 1.8)	1.4 (0.9, 2.2)
Fair/poor	1.1 (0.6, 2.0)	1.5 (0.7, 3.2)	0.6 (0.4, 0.9)	0.6 (0.4, 0.9)	0.9 (0.6, 1.4)	0.9 (0.5, 1.5)
Drug use: crack or cocaine in the past 6 months	1.4 (0.8, 2.3)	1.0 (0.5, 2.1)	0.8 (0.5, 1.1)	1.0 (0.6, 1.6)	1.1 (0.7, 1.6)	0.9 (0.6, 1.6)
Drug use: other drugs in the past 6 months^d^	2.9 (1.9, 4.6)	1.2 (0.6, 2.3)	0.9 (0.6, 1.2)	0.8 (0.5, 1.2)	1.7 (1.2, 2.4)	1.1 (0.7, 1.8)
Alcohol misuse: AUDIT-C Score ≥ 4^e^	0.9 (0.5, 1.3)	0.6 (0.3, 1.0)	0.7 (0.5, 1.0)	0.9 (0.6, 1.2)	1.2 (0.8, 1.7)	1.0 (0.7, 1.5)

*OR* odds ratio, *CI* confidence interval, *aOR* adjusted odds ratio, *STBBI* sexually transmitted or blood borne infection, *AUDIT-C* alcohol use disorders identification test, consumption questions

^a^Reference level: Class 4—low use of prevention; confidence intervals account for clustering by participant recruiter

^b^Sexual orientation was defined as gay versus other. The other terms used to describe one’s sexual orientation included: bisexual, straight, queer, questioning, asexual, pansexual and two-spirit

^c^The HIV Treatment Optimism-Skepticism Scale [62] includes items related to the efficacy of antiretrovirals for both HIV treatment and reduced infectiousness. The scale ranges from 0 to 36, where higher scores indicate higher optimism in antiretroviral treatment

^d^Other drugs included any of ecstasy, crystal methamphetamine, mephedrone, speed, poppers, gamma hydroxybutyrate (GHB), lysergic acid diethylamide (LSD), and ketamine

^e^Alcohol misuse was measured by the Alcohol Use Disorders Identification Test, consumption questions (AUDIT-C), a screening tool for alcohol abuse, dependence, or heavy drinking[63]. The AUDIT-C Scale ranges from 0 to 12, where higher scores indicate higher risk of alcohol affecting one’s health and safety. Scores were dichotomized at the optimal cut point for identifying alcohol dependence in men [64]: ≥ 4 vs. lower

#### Individuals Living with HIV

The referent class was *mostly ART with viral suppression.* The multivariable model indicates those in the *ART with viral suppression and seroadaptive behaviour use* and *ART with viral suppression and condom use* classes were more likely to report an increased number of anal sex partners (P6M) across all categories (Table 5; qualitative overview in Table S9). The *ART with viral suppression and condom use* class members were also less likely to report having used other drugs (P6M; aOR 0.2, 95% CI 0.1–0.6) and less likely to have a higher HIV Optimism-Skepticism score (aOR 0.9, 95% CI 0.8–1.0). The *ART with viral suppression and seroadaptive behaviour use* class members were more likely to have a post-high school diploma or higher (aOR 2.4, 95% CI 1.0–5.5).

**Table 5:** Univariable and multivariable multinomial logistic regression model results assessing factors associated with latent class membership among the HIV-positive participants of the Engage-Montreal study, 2017–2018 (n = 200)^a,b^

Variable	Class 2: ART with viral suppression and condom use		Class 3: ART with viral suppression and seroadaptive behaviour use	
	OR (95% CI)	aOR (95% CI)	OR (95% CI)	aOR (95% CI)
Age ≤ 50	0.8 (0.4, 1.8)	1.9 (0.5, 6.4)	2.2 (1.2, 4.2)	1.5 (0.6, 3.6)
Sexual orientation: gay^c^	1.4 (0.5, 4.0)	1.9 (0.3, 11.7)	2.0 (0.7, 5.7)	1.4 (0.5, 4.5)
Ethnicity^d^				
French Canadian	1.00 (referent)	1.00 (referent)	1.00 (referent)	1.00 (referent)
English Canadian	0.5 (0.1, 1.8)	1.1 (0.2, 5.3)	0.8 (0.3, 2.3)	0.6 (0.2, 2.2)
Other	1.3 (0.6, 2.8)	2.2 (0.8, 5.9)	1.0 (0.4, 2.3)	0.5 (0.2, 1.4)
Education: post-high school diploma or higher	0.6 (0.3, 1.3)	0.5 (0.2, 1.3)	2.4 (1.3, 4.6)	2.4 (1.0, 5.5)
Social time spent with gay/bi guys: 50% or more	1.0 (0.5, 2.0)	1.0 (0.4, 2.5)	1.5 (0.8, 2.7)	1.1 (0.5, 2.5)
STBBI diagnosis in the past 12 months	0.5 (0.2, 0.9)	0.5 (0.2, 1.4)	1.8 (0.9, 3.4)	0.7 (0.3, 1.6)
Number of anal sex partners in the past 6 months				
0–1	1.00 (referent)	1.00 (referent)	1.00 (referent)	1.00 (referent)
2–3	2.0 (0.8, 5.5)	3.1 (0.8, 12.2)	8.6 (2.0, 36.2)	10.2 (1.6, 63.5)
4+	0.9 (0.4, 2.1)	2.9 (0.8, 11.4)	13.3 (4.1, 42.9)	15.0 (3.1, 73.5)
Currently have a main partner	0.8 (0.4, 1.6)	0.5 (0.2, 1.5)	0.6 (0.3, 1.2)	0.6 (0.2, 1.4)
HIV Optimism-Skepticism Scale^e^	0.9 (0.8, 0.9)	0.9 (0.8, 1.0)	1.0 (1.0, 1.1)	1.0 (0.9, 1.1)
Perceived mental health in the past 6 months				
Excellent/very good	1.00 (referent)	1.00 (referent)	1.00 (referent)	1.00 (referent)
Good	0.6 (0.3, 1.3)	0.5 (0.2, 1.3)	1.2 (0.5, 2.5)	0.8 (0.3, 2.2)
Fair/poor	0.4 (0.1, 1.0)	0.3 (0.1, 1.2)	1.4 (0.7, 2.8)	0.9 (0.3, 2.6)
Drug use: crack or cocaine in the past 6 months	0.6 (0.2, 1.4)	0.8 (0.2, 3.0)	1.0 (0.5, 2.2)	1.0 (0.4, 2.5)
Drug use: other drugs in the past 6 months^f^	0.3 (0.1, 0.6)	0.2 (0.1, 0.6)	3.4 (1.6, 7.3)	1.3 (0.5, 3.3)
Alcohol misuse: AUDIT-C Score ≥ 4^g^	0.5 (0.3, 1.1)	0.6 (0.2, 1.4)	1.0 (0.5, 1.8)	0.9 (0.4, 1.9)

*ART* antiretroviral treatment, *OR* odds ratio, *CI* confidence interval, *aOR* adjusted odds ratio, *STBBI* sexually transmitted or blood borne infection, *AUDIT-C* alcohol use disorders identification test, consumption questions

^a^Reference level: Class 1—mostly ART with viral suppression; confidence intervals account for clustering by participant recruiter

^b^The following variables could not be assessed in univariable or multivariable multinomial regression models due to small cell counts: HIV status of current main partner, perceived risk of transmitting HIV, and currently have a health care provider

^c^Sexual orientation was defined as gay versus other. The other terms used to describe one’s sexual orientation included: bisexual, straight, queer, questioning, asexual, pansexual and two-spirit

^d^Ethnicity is redefined in these models as French Canadian, English Canadian, and other (including European) due to small cell counts

^e^The HIV Treatment Optimism-Skepticism Scale [62] includes items related to the efficacy of antiretrovirals for both HIV treatment and reduced infectiousness. The scale ranges from 0 to 36, where higher scores indicate higher optimism in antiretroviral treatment

^f^Other drugs included any of ecstasy, crystal methamphetamine, mephedrone, speed, poppers, gamma hydroxybutyrate (GHB), lysergic acid diethylamide (LSD), and ketamine

^g^Alcohol misuse was measured by the Alcohol Use Disorders Identification Test, consumption questions (AUDIT-C), a screening tool for alcohol abuse, dependence, or heavy drinking [63]. The AUDIT-C Scale ranges from 0 to 12, where higher scores indicate higher risk of alcohol affecting one’s health and safety. Scores were dichotomized at the optimal cut point for identifying alcohol dependence in men [64]: ≥ 4 vs. lower

## Discussion

The combination of prevention strategies targeting different transmission pathways is our best option to sustainably reduce HIV incidence. We assessed combination prevention at the individual level, where strategies practiced by GBM might vary within and across sexual partners or experiences [14, 69]. In Montreal, we found condoms remain a preferred strategy used by many GBM, but antiretroviral-based prevention methods are now distinctly reported. The proportion of GBM who are living with HIV and aware of their status with a suppressed viral load was very high for all combination prevention classes (84–93%), indicating diagnosed GBM living with HIV in Montreal are being engaged into HIV care. However, we also observed that 9% of GBM reported not knowing if they were HIV-negative or -positive, underscoring the importance of reaching those GBM for HIV testing, as diagnosing those unaware they are living with HIV is essential for treatment as prevention to be effective. These testing encounters could also be an opportunity for health care providers to discuss current prevention strategies with GBM. Among HIV-negative/unknown GBM, an estimated 7% belonged to the class adopting *biomedical prevention*, 84% of which reported PrEP use. Yet, among HIV-negative GBM overall, this level of PrEP coverage may be too low to sustainably reduce HIV incidence [70–72]. In general, the patterns we observed suggest that within classes of prevention users, especially in HIV-negative GBM, the combining of different prevention types was limited. Ensuring both HIV-negative GBM and GBM living with HIV are aware of and have access to many of today’s tools would allow them to determine which strategies will meet their needs in different situations. This could increase the adoption of multiple strategies and the individual-level use of combination prevention. Further, for combination prevention to be effective at the population-level, matching appropriate strategies to the risk profiles of GBM must be promoted. Increasing healthcare provider awareness of and sensitivity to these profiles would also be essential, to assist in their approach to patient engagement in preventive care and encourage appropriate conversations around U = U and PrEP.

Correlates of class membership suggest HIV-negative/unknown GBM with an increased number of anal sex partners or reported STBBI diagnosis are more commonly in classes of *biomedical prevention use, condom use*, and *seroadaptive behaviour use*. For the *condom use *class, in particular, the association with an STBBI diagnosis presents an apparent discordance, despite the uncertainty in this estimate; however, it is possible that receiving an STBBI diagnosis led to the use of condoms consistently. Those in the remaining subgroup, who use fewer prevention strategies, are ultimately at a lower risk of HIV acquisition. Among this class, 27% of GBM reported not having any anal sex partners in the P6M, and 33% had only one. Despite this, the risk among GBM in this group is not necessarily null and some might be missed in the reach of current prevention programs. The *biomedical prevention use* class had higher optimism in ART and reported feeling less at risk for HIV acquisition while being more likely to have a main partner whose HIV-status is positive, suggesting a higher prevention awareness among these HIV-negative GBM, perhaps through their experience of having a partner that is living with HIV. This may also be explained by the fact these men were more likely to have attained a higher level of education, possibly having a higher level of health literacy as well. Community-based promotion of antiretroviral-based prevention could, therefore, be needed to inform GBM of their effective protection against HIV transmission more widely. Among GBM living with HIV, we similarly identified subgroups of *ART with viral suppression and condom use* and *ART with viral suppression and seroadaptive behaviour use* more likely to have an increased number of anal sex partners. We also observed a subgroup using biomedical prevention (*ART with viral suppression*) alone. Members of this class did have a high probability of a suppressed viral load, suggesting U = U is appropriately being practiced by this class; however, whether these GBM explicitly consider their use of ART as a prevention strategy is not known.

Our results are generally consistent with the previous Montreal study that identified subgroups of HIV-negative/unknown GBM adopting condoms and various seroadaptive strategies [32]. Our analyses, however, suggest the emergence of a new class of combination prevention among HIV-negative/unknown GBM using antiretroviral-based prevention strategies, especially PrEP. Notably, members of this class had a somewhat low probability of using condoms consistently (34%), in-line with indications for initiating PrEP in Quebec (condomless anal sex in the P6M and one additional risk factor for HIV acquisition) [73], as well as the known efficacy of PrEP, and supported by findings from a clinical cohort of PrEP users in Montreal [74]. PrEP can also be among the strategies GBM living with HIV might use—they can perform PrEP sorting, whereby they consider the PrEP-status of HIV-negative GBM when choosing such sexual partners [75]. In our study, we also saw a high proportion of GBM living with HIV adopting seroadaptive behaviours by choosing to have condomless anal sex with GBM living with HIV or HIV-negative GBM on PrEP. Neither these, nor PrEP use, however, prevent the transmission of other STBBIs and their utilization alone could have implications on their spread within GBM [76, 77], especially as these sub-groups had a low probability of condom use.

Studies elsewhere also used LCA to investigate prevention use among various populations of GBM. These mainly focused on serosorting and seropositioning [33, 42, 43]. While these are well known to be practiced by GBM across various settings [11–17], their level of use is expected to differ in our study, given the additional strategies we assessed and our consideration of viral load and PrEP sorting. Further, the use of seroadaptive practices could be lower among antiretroviral medication users. Only Dangerfield et al. [42] assessed seroadaptive strategies in a time of PrEP availability. Qualitatively similar to our results, this study found the majority of Paris GBM belonged to a class defined by a low number of condomless anal sex acts. To our knowledge, few other studies of GBM have assessed such a variety of biomedical HIV prevention strategies [77–80]. Studies conducted in France, Australia, and the United States assessed time trends in prevention use by GBM [77–79]. In line with our findings, all observed that, despite decreases, consistent condom use remained a dominant strategy in recent years, and the use of antiretroviral-based strategies was increasing [77–79]. Regarding biomedical seroadaptive behaviours, Grov et al. [80] also considered these and witnessed PrEP and viral load sorting taking place amongst GBM in the United States.

This study has several limitations. First, like previous studies on this topic [32, 33], our results are limited by self-reported measures, which could be influenced by a social desirability bias and imperfect recall. This bias could lead to an overestimation of any prevention strategy use; however, use of a computer-assisted questionnaire likely helped mitigate this [81]. Second, the nature of prevention is dynamic, with an individual’s adoption of various strategies changing over time, based on their perceived risk of HIV acquisition or transmission. Using a recall period of the P6M for many of the prevention indicators may be too limited to capture this reality entirely. A longitudinal assessment of combination prevention is needed, which Engage could perform in the future. Third, given the cross-sectional nature of our RDS survey, temporality between measures cannot be inferred. For instance, we cannot claim whether the low proportion having used condoms in the P6M is a reason for initiating, or a consequence of, PrEP use. Fourth, we considered recreational drug and alcohol use in our analyses. However, situational use of drugs or alcohol during sex are also important factors related to use of prevention, and these were not assessed. Lastly, as an exploratory analysis investigating many correlates of class membership, the precision of the regression estimates was reduced. Future analyses including participants from all Engage sites would likely improve the precision of estimates and would allow for an examination of whether classes vary by city.

The study strengths include the large sample of GBM and method of recruitment, RDS, which may improve its representativeness. The comprehensive study questionnaire enabled the collection of information on several relevant biomedical and behavioural prevention strategies. Again, the results we observed are consistent with the previous work in Montreal, with the emergence of a new class among HIV-negative/unknown GBM being plausible given the evolving nature of prevention and its accessibility. Importantly, eliminating HIV will not be achieved without fully appreciating transmission dynamics and the need for prevention use by both HIV-negative and GBM living with HIV. In this study, we viewed prevention broadly, adopting an HIV status-neutral approach and assessing prevention strategies acting on both HIV transmission and acquisition.

## Conclusions

The number of HIV diagnoses among GBM in Canada has remained relatively stable in recent years. Achieving the UNAIDS Fast-Track city targets in Montreal will require the scale-up of combination HIV prevention strategies meeting the needs of both HIV-negative GBM and GBM living with HIV. With combination prevention, individuals identify the HIV prevention strategies best suited for them. This LCA of combination prevention is the first to include PrEP use and, indeed, demonstrated its emergence as a distinct prevention strategy used by Montreal GBM. Our finding that the HIV-negative/unknown GBM currently using biomedical prevention are those with a higher level of formal education is important. This observation, in conjunction with biomedical prevention use being the smallest class of HIV-negative/unknown GBM, indicates the need to continue to raise awareness of PrEP’s effectiveness and to promote its availability for HIV at-risk GBM. Moreover, despite medication reimbursement for PrEP in Quebec, out-of-pocket costs (up to $93/month) could be a barrier to PrEP access which should be further assessed. Identifying sub-groups of GBM highly vulnerable to HIV transmission and tailoring appropriate combination prevention programs to their needs will also be important.

Future work should use longitudinal data to assess the consistency of these results and monitor potential variability in prevention over time. The implications of HIV prevention strategies on STI transmission in Montreal should also be examined, particularly among those using mainly seroadaptive behaviours or PrEP, as GBM may not be consistently using condoms when utilizing these measures.

## Electronic supplementary material

Below is the link to the electronic supplementary material.

Supplementary file1 (DOCX 46 kb)
