# Supplementary material for: Combination HIV Prevention Strategies Among Montreal Gay, Bisexual, and Other Men Who Have Sex with Men in the PrEP Era: A Latent Class Analysis
Source: AIDS Behav. 2020 Jul 9;25(1):269–83. doi: 10.1007/s10461-020-02965-4 (PMC7846508; doi:10.1007/s10461-020-02965-4)
Supplement: Supplementary file 1 — Supplementary file1 (DOCX 46 kb) [file 10461_2020_2965_MOESM1_ESM.docx]

**Supplementary Material**

**Appendix I: Additional details on the use of respondent-driven sampling (RDS) in the 2017-2018 Engage-Montreal study, according to the Strobe-RDS guidelines (1)**

*Study setting*

The primary purpose of the Engage study is to have an updated understanding of the sexual health of gay, bisexual, queer and other men who have sex with men (GBM), including transmen, in order to support HIV and other sexually transmitted or blood-borne infection (STBBI) prevention efforts (2). The Engage study utilized RDS to obtain a representative sample of GBM at each study site (Montreal, Toronto, and Vancouver). In Montreal, participant recruitment and data collection took place between February 2017–June 2018 at a community-based study site.

*Formative research findings*

Prior to study initiation, formative research involving engagement and consultation with local community organizations and individual community members was carried out by the study investigators. This formative work aided in identifying a diverse group of study seeds, validating study procedures, and creating promotional materials.

*Seed selection*

With RDS, initial participants (or ‘seeds’) are purposively selected such that they are eligible for the study, socially connected to many members of the population, and altogether characterize diverse features of the population in order to access its various subgroups (3). In Engage, selected seeds were required to self-report as motivated to recruit additional participants into the study and know a minimum of six other GBM that would satisfy the study eligibility criteria. To obtain a diverse sample, 25 seeds of varying age, HIV status, gender identity, and ethnicity were initially selected. Monitoring of the participant sample composition was performed throughout the recruitment and data collection period, which allowed the study investigators to assess the progress of the RDS recruitment and potential need for additional seeds. Throughout recruitment in Montreal, two additional seeds were added in order to capture younger GBM in the study sample.

*RDS recruitment*

The process of RDS recruitment was explained to all participants by an onsite study staff member (either a research assistant or study nurse). Beginning with the seeds, participants were provided with six coupons to distribute to eligible and interested GBM that had not already participated in the study. Those who did participate were compensated with $50, as well as $15 for each successful recruit. The option to forego the $50 and instead enter into a draw for a $250 prepaid credit card or a $2000 travel voucher was also provided to all participants. In general, there were no time limits set for referrals; however, in the last two months of recruitment (April-May 2018), participants distributing coupons were informed that the recruitment period would end by June 2018. Those who participated in the last three weeks of the recruitment period were not provided with any invitation coupons.

*Eligibility*

All potential participants were screened over the phone or onsite for eligibility by a research coordinator or study nurse. Study eligibility criteria included:

Gender identify as a man, including transgender man

≥16 years

Had sex with another man in the past six months

Reside in the greater Montreal area, and

Able to read in French or English.

*Participant database*

A participant database containing information on each participant, including their name, contact information, study identification number, referrer identification number, and coupon numbers (for each of the six coupons issued) was established. This database was monitored and updated continuously to ensure that repeat enrollments did not occur.

*Study sample*

Engage aimed to enroll 1000 participants at the Montreal site. Upon completion of the recruitment process a total of 1179 eligible GBM had participated in Montreal. The number of coupons issued and returned were 6822 and 1152, respectively.

*Self-reported personal network size*

The following question was used to obtain information on participant personal network size in the Engage questionnaire: “*How many men who have sex with men aged 16 years or older, including trans men, do you know who live or work in the [Metro Vancouver/Greater Toronto/Metro Montreal depending on site] area (whether they identify as gay or otherwise)? This includes gay/bi guys you see or speak to regularly; e.g., close friends, boyfriends, spouses, regular sex partners, roommates, relatives, people you regularly hang out with, etc.”*

*Recruiter-recruit relationship*

Unique identification numbers were assigned to each participant, and the information on who recruited whom was tracked by recording the recruiter’s identification number in the study participant database.

*Study Analyses*

Study seeds were included in all analyses. In this study, RDS adjustments of the sociodemographic characteristics and latent class sizes were made using the RDS-II estimator. This estimator weights the sample proportions by the inverse of the self-reported participant network size.

**Appendix II: Survey questions corresponding to the self-reported measures of combination HIV prevention methods examined in this study.**

| **Table S1: Survey items from the 2017-2018 Engage study questionnaire used to measure HIV prevention methods according to HIV status** | |
| --- | --- |
| **Prevention Method^a^** | **Survey Question** |
| **HIV-negative/unknown** | |
| Recent HIV testing^b^ | *When were you last tested for HIV?* |
| PEP use^b^ | *Have you ever taken PEP?* |
| PrEP use^b^ | *Have you ever taken PrEP yourself?* |
| Consistent condom use^b^ | *Some HIV negative guys use strategies to prevent getting HIV. Have you done any of the following to prevent getting HIV in the past 6 months?* $\to$ ***Always used condoms for anal sex*** |
| Strategic positioning^b^ | *Some HIV negative guys use strategies to prevent getting HIV. Have you done any of the following to prevent getting HIV in the past 6 months?* $\to$ ***Being the top (insertive partner) for anal sex*** |
| Serosorting | *Some HIV negative guys use strategies to prevent getting HIV. Have you done any of the following to prevent getting HIV in the past 6 months?* $\to$ ***Had sex without condoms with guys I know are HIV-negative*** |
| Viral load sorting | *Some HIV negative guys use strategies to prevent getting HIV. Have you done any of the following to prevent getting HIV in the past 6 months?* $\to$ ***Had sex without condoms with HIV-positive guys who have 'undetectable' (low) viral loads*** |
| Serosorting or viral load sorting^b^ | *As above.* |
| **HIV-positive** | |
| ART with viral suppression^c^ | *AT THIS TIME, what do you think your HIV viral load is?*  $\to Undetectable (>$*50 copies/mL)* |
| Consistent condom use^c^ | *Some HIV-positive guys use strategies to prevent transmitting HIV to their sex partners. Have you done any of the following to prevent your sex partners from getting HIV in the past 6 months?*  $\to$ ***Always used condoms for anal sex*** |
| Strategic positioning^c^ | *Some HIV-positive guys use strategies to prevent transmitting HIV to their sex partners. Have you done any of the following to prevent your sex partners from getting HIV in the past 6 months?*  $\to$ ***Being the bottom (receptive partner) for anal sex*** |
| Serosorting | *Some HIV-positive guys use strategies to prevent transmitting HIV to their sex partners. Have you done any of the following to prevent your sex partners from getting HIV in the past 6 months?*  $\to$ ***Had sex without condoms with guys I know are HIV-positive*** |
| PrEP-use sorting | *Some HIV-positive guys use strategies to prevent transmitting HIV to their sex partners. Have you done any of the following to prevent your sex partners from getting HIV in the past 6 months?*  $\to$ ***Had sex without condoms with HIV-negative guys who are using PrEP*** |
| Serosorting and/or PrEP-use sorting^c^ | *As above.* |

Abbreviations: Post-exposure prophylaxis (PEP); pre-exposure prophylaxis (PrEP); antiretroviral treatment (ART).

^a^Use of each prevention method was measured in the past 6 months, except for PEP (ever use) and ART with viral suppression (current).

^b^Used as an indicator in HIV-negative/unknown latent class analysis models.

^c^Used as an indicator in HIV-positive latent class analysis models.

**Appendix III: Additional tables of study results**

| **Table S2: Model fit statistics of all latent class models performed on self-reported use of HIV prevention methods among the HIV-negative/unknown participants of the Engage-Montreal study, 2017 – 2018 (n=968)** | | | | | | |
| --- | --- | --- | --- | --- | --- | --- |
| **Model** | **Log-likelihood** | **Residual df** | **BIC** | **AIC** | **LR** | **Entropy** |
| 1 class | -3366.955 | 57 | 6775.161 | 6745.909 | 445.517 | 3.48 |
| 2 class | -3211.355 | 50 | 6512.088 | 6448.710 | 134.318 | 3.33 |
| 3 class | -3180.187 | 43 | 6497.878 | 6400.373 | 71.981 | 3.30 |
| 4 class | -3161.094 | 36 | 6507.820 | 6376.189 | 33.797 | 3.27 |
| 5 class | -3155.503 | 29 | 6544.765 | 6379.007 | 22.615 | 3.26 |

Abbreviations: Degrees of freedom (df); Bayes Information Criterion (BIC); Akaike Information Criterion (AIC); likelihood ratio (LR)

| **Table S3: Bivariate residuals between item pairs of self-reported use of HIV prevention methods among the HIV-negative/unknown participants of the Engage-Montreal study, 2017 – 2018 (n=968): 4 class model** | | | | | |
| --- | --- | --- | --- | --- | --- |
| **Variable** | HIV testing | PrEP | PEP | Condom | Strategic positioning |
| HIV testing |  |  |  |  |  |
| PrEP | 0.33 |  |  |  |  |
| PEP | 0.05 | 0.05 |  |  |  |
| Condom | 0.02 | 0.09 | 0.09 |  |  |
| Strategic positioning | 0.02 | 0.05 | 0.18 | 1.02 |  |
| Serosorting or viral load sorting | 0.03 | 0.10 | 0.10 | 0.55 | 0.20 |

Abbreviations: Pre-exposure prophylaxis (PrEP); post-exposure prophylaxis (PEP).

| **Table S4: Unadjusted and RDS-II adjusted class sizes of the 4-class model of self-reported use of HIV prevention methods among the HIV-negative/unknown participants of the Engage-Montreal study, 2017 – 2018 (n=968)^a^** | | |
| --- | --- | --- |
| **Class** | **Unweighted estimate** | **RDS-II weighted estimate (95% CI)** |
| 1: Biomedical prevention use | 12% | 7% (4-10%) |
| 2: Condom use | 35% | 40% (34-45%) |
| 3: Seroadaptive behaviour use | 25% | 21% (17-26%) |
| 4: Low use of prevention | 28% | 32% (27-37%) |

Abbreviations: Respondent driven sampling weights (RDS-II); confidence interval (CI).

^a^RDS-II weights are inverse probability of sampling weights that are proportional to participant network size.

| **Table S5: Model fit statistics of all latent class models performed on self-reported use of HIV prevention methods among the HIV-positive participants of the Engage-Montreal study, 2017 – 2018 (n=200)** | | | | | | |
| --- | --- | --- | --- | --- | --- | --- |
| **Model** | **Log-likelihood** | **Residual df** | **BIC** | **AIC** | **LR** | **Entropy** |
| 1 class | -547.972 | 25 | 1127.733 | 1107.943 | 61.564 | 21.92 |
| 2 class | -532.371 | 18 | 1133.620 | 1090.742 | 30.363 | 21.28 |
| 3 class | -524.167 | 11 | 1154.300 | 1088.333 | 13.954 | 20.98 |

Abbreviations: Degrees of freedom (df); Bayes Information Criterion (BIC); Akaike Information Criterion (AIC); likelihood ratio (LR)

| **Table S6: Bivariate residuals between item pairs of self-reported use of HIV prevention methods among the HIV-positive participants of the Engage-Montreal study, 2017 – 2018 (n=200): 3 class model** | | | |
| --- | --- | --- | --- |
| **Variable** | Viral suppression | Condom | Strategic positioning |
| Viral suppression |  |  |  |
| Condom | 1.40 |  |  |
| Strategic positioning | 1.44 | 0.35 |  |
| Serosorting and/or PrEP sorting | 0.78 | 0.26 | 0.39 |

Abbreviations: Pre-exposure prophylaxis (PrEP)

| **Table S7: Unadjusted and RDS-II adjusted sizes of the 3-class model of self-reported use of HIV prevention methods among the HIV-positive participants of the Engage-Montreal study, 2017 – 2018 (n=200)^a^** | | |
| --- | --- | --- |
| **Class** | **Unweighted estimate** | **RDS-II weighted estimate (95% CI)** |
| 1: Mostly ART with viral suppression | 44% | 53% (41-65%) |
| 2: ART with viral suppression and condom use | 23% | 19% (16-41%) |
| 3: ART with viral suppression and seroadaptive behaviour use | 34% | 18% (10-27%) |

Abbreviations: Respondent driven sampling weights (RDS-II); confidence interval (CI); antiretroviral treatment (ART).

^a^RDS-II weights are inverse probability of sampling weights that are proportional to participant network size.

| **Table S8: Overview of multivariable multinomial logistic regression model results assessing factors associated with latent class membership among the HIV-negative/unknown participants of the Engage-Montreal study, 2017 – 2018 (n=968)^a^** | | |
| --- | --- | --- |
| **Class 1:**  **Biomedical prevention use** | **Class 2:**  **Condom use** | **Class 3:**  **Seroadaptive behaviour use** |
| ⎻ | ↑ European ethnicity | ↑ European ethnicity^b^ |
| ⎻ | ↑ Other ethnicity | ⎻ |
| ↑ Education | ⎻ | ⎻ |
| ⎻ | ⎻ | ↑ Spend 50% or more of social time with gay/bi guys^b^ |
| ⎻ | ⎻ | ↓ Unaware of HIV status^c^ |
| ↑ STBBI diagnosis in past 12 months | ↑ STBBI diagnosis in last 12 months^b^ | ↑ STBBI diagnosis in past 12 months |
| ↑ 2-3 anal sex partners in past 6 months | ↑ 2-3 anal sex partners in past 6 months | ↑ 2-3 anal sex partners in past 6 months |
| ↑ 4-5 anal sex partners in past 6 months | ↑ 4-5 anal sex partners in past 6 months | ↑ 4-5 anal sex partners in past 6 months |
| ↑ 6+ anal sex partners in past 6 months | ↑ 6+ anal sex partners in past 6 months | ↑ 6+ anal sex partners in past 6 months |
| ↓ Have a main partner they were certain was HIV-negative^c^ | ↓ Have a main partner they were certain was HIV-negative | ⎻ |
| ↑ Have a main partner they were certain was HIV-positive^b^ | ⎻ | ⎻ |
| ↓ Perceived risk of HIV^c^ | ⎻ | ⎻ |
| ↑ score on HIV Optimism-Skepticism Scale^b^ | ⎻ | ⎻ |
| ↑ Health care provider | ⎻ | ⎻ |
| ⎻ | ↓ Report in fair/poor mental health in the past 6 months | ⎻ |
| ↓ Alcohol misuse^c^ | ⎻ | ⎻ |

Abbreviations: Sexually transmitted or blood borne infection (STBBI)

^a^Reference level: Class 4 – Low use of prevention

^b^An imprecise association, yet indicative of an increased odds.

^c^An imprecise association, yet indicative of a decreased odds.

| **Table S9: Overview of multivariable regression multinomial logistic model results assessing factors associated with latent class membership among the HIV-positive participants of the Engage-Montreal study, 2017 – 2018 (n=200)^a^** | |
| --- | --- |
| **Class 2: ART with viral suppression and condom use** | **Class 3: ART with viral suppression and seroadaptive behaviour use** |
| ⎻ | ↑ Education**^b^** |
| ↓ STBBI diagnosis in past 12 months^c^ | ⎻ |
| ↑ 2-3 anal sex partners in past 6 months^b^ | ↑ 2-3 anal sex partners in past 6 months |
| ↑ 4+ anal sex partners in past 6 months^b^ | ↑ 4+ anal sex partners in past 6 months |
| ↓ HIV Optimism-Skepticism score^c^ | ⎻ |
| ↓ Use of other drugs in past 6 months | ⎻ |

Abbreviations: Antiretroviral treatment (ART); sexually transmitted or blood borne infection (STBBI)

^a^Reference level: Class 1 – Mostly ART with viral suppression.

^b^An imprecise association, yet indicative of an increased odds.

^c^An imprecise association, yet indicative of a decreased odds.

**References**

1. White RG, Hakim AJ, Salganik MJ, Spiller MW, Johnston LG, Kerr L, et al. Strengthening the Reporting of Observational Studies in Epidemiology for respondent-driven sampling studies: "STROBE-RDS" statement. Journal of clinical epidemiology. 2015;68(12):1463-71.

2. Lambert G, Cox J, Messier-Peet M, Apelian H, Moodie EEM, and the members of the Engage research team. Engage Montréal, Portrait of the sexual health of men who have sex with men in Greater Montréal, Cycle 2017-2018, Highlights. Direction régionale de santé publique, CIUSSS du Centre-Sud-de-l’Île-de-Montréal; 2019 January.

3. World Health Organization. Introduction to HIV/AIDS and sexually transmitted infection surveillance: Module 4. Supplement: a guide to using RDS analyst and NetDraw. World Health Organization. Regional Office for the Eastern Mediterranean; 2014.
